# The prognostic value of major facilitator superfamily domain-containing protein 2A in patients with hepatocellular carcinoma

**DOI:** 10.18632/aging.102333

**Published:** 2019-10-04

**Authors:** Shan Xing, Jun Kan, Aishan Su, Qiao-Dan Liu, Kailin Wang, Xiuyu Cai, Jun Dong

**Affiliations:** 1 Department of Laboratory, Sun Yat-sen University Cancer Center, State Key Laboratory of Oncology in South China, Collaborative Innovation Center for Cancer Medicine, Guangzhou 510060, P. R. China; 2 Department of Oncology, The First Affiliated Hospital of Guangzhou University of Chinese Medicine, Guangzhou University of Chinese Medicine, Guangzhou 510006, China; 3 Department of GCP Center, Nanfang Hospital of Southern Medical University, Guangzhou 510515, P.R. China; 4 Department of Radiation Oncology, The Fifth Affiliated Hospital of Sun Yat-sen University, Zhuhai 519001, Guangdong Province, China; 5 Department of Oncology, The First Affiliated Hospital of Guangdong Pharmaceutical University, Guangdong Pharmaceutical University, Guangzhou 510062, China; 6 Department of Integrated Therapy in Oncology, Sun Yat-sen University Cancer Center, State Key Laboratory of Oncology in South China, Collaborative Innovation Center for Cancer Medicine, Guangzhou 510060, P. R. China

**Keywords:** MFSD2A, hepatocellular carcinoma, poor differentiation, prognosis

## Abstract

Introduction: We aimed to characterize the expression of major facilitator superfamily domain-containing protein 2A (MFSD2A) in hepatocellular carcinoma (HCC) patients and analyze its prognostic value.

Results: Immunohistochemistry revealed that low expression of MFSD2A was present in 37 of 79 cases (46.84%), which was significantly correlated with poor histological differentiation (*P* = 0.012). The plasma MFSD2A level in HCC patients was significantly lower than in healthy controls (*P* = 0.0079) and controls with chronic hepatitis B virus (HBV) infection (*P* = 0.0430). Moreover, patients with lower MFSD2A expression had shorter survival than higher expression (*P* = 0.021). Multivariate analysis revealed that MFSD2A was an independent prognostic predictor for HCC patients (*P* = 0.027).

Conclusion: The current study indicate MFSD2A may be an optimal diagnostic and prognostic biomarker for HCC.

Methods: First, we examined MFSD2A expression in 24 paired HCC and nontumorous tissues by real-time quantitative PCR (RT-qPCR). Second, the protein levels of MFSD2A in 11 paired HCC and nontumorous tissues were investigated by western blotting (WB). Moreover, MFSD2A protein expression was evaluated by immunohistochemistry in 79 HCC patients. In addition, we detected the plasma level of MFSD2A in HCC patients and healthy individuals and investigated the relationship between MFSD2A expression and clinicopathological parameters or prognosis of HCC patients.

## INTRODUCTION

Hepatocellular carcinoma (HCC) is one of the most common cancer types and the third leading cause of cancer-related deaths worldwide, affecting one million individuals annually [1]. More importantly, the incidence of HCC is still increasing worldwide [2]. Despite improvements in treatment strategies, the mortality rate remains high because of the lack of sensitive and specific markers for HCC. Alpha-fetoprotein (AFP) has been widely used as a biomarker for HCC, not only in diagnosis but also in surveillance [3–5]. However, one study reported that the sensitivity of AFP in elderly HCC patients was approximately 41.0%, while another study demonstrated it to be 60.5% [6, 7]. Previous studies have shown that the efficiency for predicting the prognosis of HCC using serum AFP levels remains unsatisfactory (30–62%). In addition, the dynamic AFP levels range varies from one patient to another [8]. Therefore, the application of AFP is still limited in practice. The discovery of a marker for early diagnosis, as well as prognostic prediction, in HCC is urgent.

Major facilitator superfamily domain-containing protein 2A (MFSD2A) is a membrane protein for transporting various substrates, such as sugars, polyols, drugs, and neurotransmitters, although most members of this protein family are substrate-specific [9, 10]. MFSD2A expression has been reported in many tissues, especially the liver, and is previously known to maintain blood-brain barrier function [11]. Recently, MFSD2A has been reported as a novel tumor suppressor gene that plays an important role in modulating the cell cycle and matrix attachment [12]. Another study also showed that MFSD2A+ hepatocytes repopulate the liver during injury and regeneration [13]. In China, most HCC cases result from chronic hepatitis B and are often associated with injury and regeneration of hepatocytes. Therefore, we hypothesize that MFSD2A may be another biomarker for the diagnosis and progression of HCC. However, there is no relative study of MFSD2A in HCC. Here, we investigated the expression of MFSD2A in HCC patients and analyzed its correlation with survival.

## RESULTS

### Low expression of MFSD2A in HCC tissues

**Figure 1:**
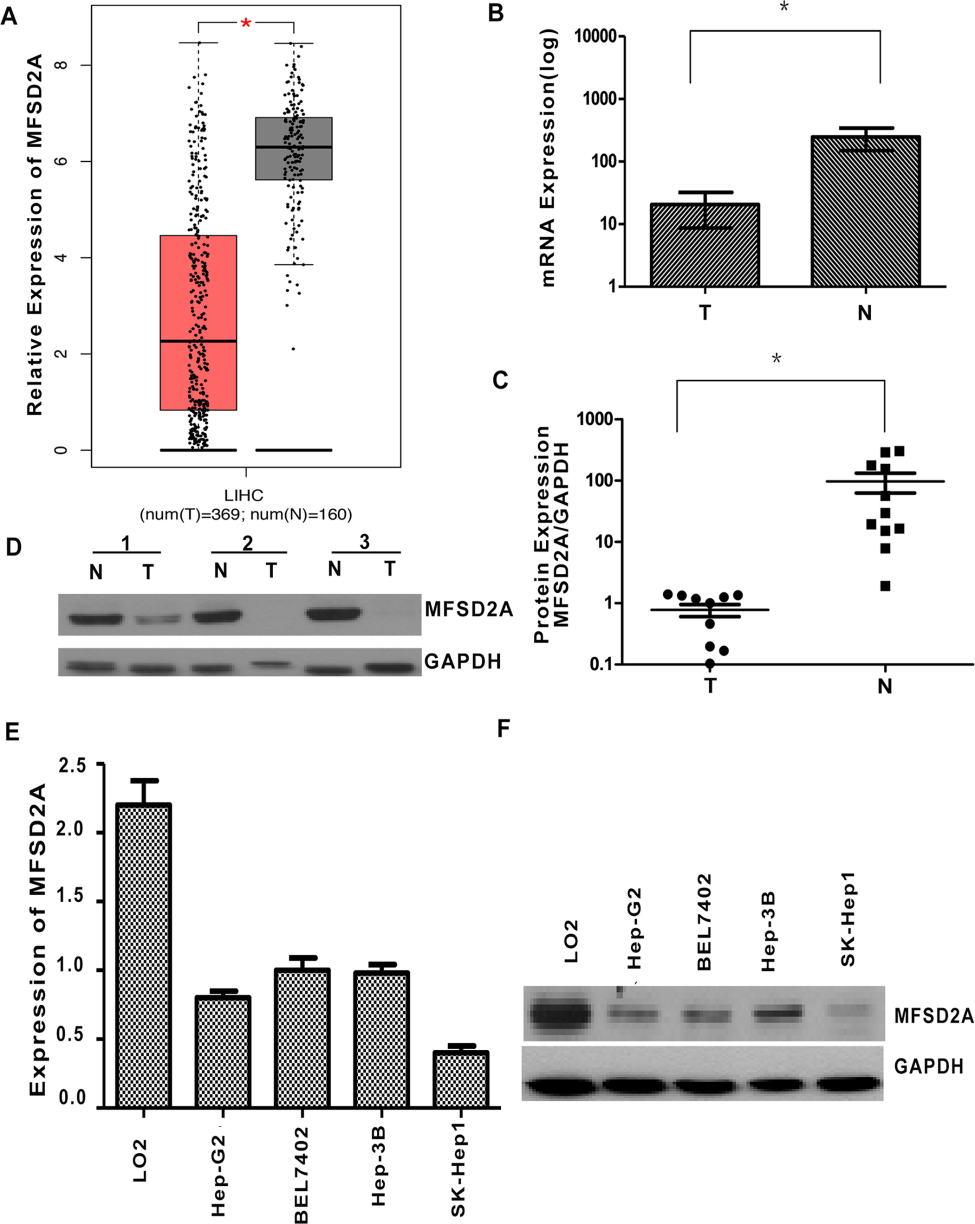
**Decreased expression of MFSD2A in HCC.** (**A**) The expression of MFSD2A in HCC and normal liver tissues was analyzed in the TCGA and GTEx databases (*P* < 0.01). (**B**) RT-qPCR showed that the relative mRNA expression of MFSD2A in HCC tissues was decreased compared with that in the matched adjacent nontumorous tissues (n = 24, *P = 0.016). (**C**) Densitometric analysis of MFSD2A protein levels relative to GAPDH in HCC and corresponding normal liver samples. The expression of MFSD2A was reduced in tumor tissues when compared with that in corresponding nontumorous tissues (n = 11, *P = 0.0472). (**D**) The protein level of MFSD2A in HCC and corresponding nontumorous specimens was tested by western blotting. GAPDH was used as a loading control. RT-qPCR (**E**) and western blotting (**F**) were used to analyze the expression of MFSD2A in several HCC cell lines and one immortalized hepatic cell line LO2.

The expression of MFSD2A in HCC and normal liver tissues was first analyzed in the TCGA and GTEx databases using the GEPIA web server [14]. The results showed significantly lower expression of MFSD2A in HCC tissues than in normal liver tissues (*P* < 0.01, Figure 1A). To confirm the expression of MFSD2A in public databases, we collected a total of 35 paired HCC and matched adjacent noncancerous tissues. Fresh tissue samples were frozen in liquid nitrogen and stored at -80°C. The transcript levels of MFSD2A were determined by RT-qPCR in 35 paired HCC and matched adjacent noncancerous tissues. The mRNA level of MFSD2A was significantly lower in 19 cancerous tissues (79%) than in the matched adjacent noncancerous tissues (*P* = 0.016, Figure 1B). We also tested MFSD2A levels in HCC samples by western blotting. The expression of MFSD2A was significantly decreased in cancerous tissues relative to that in the matched adjacent normal tissues (*P* = 0.0472, Figure 1C, 1D), which was consistent with the RT-qPCR and public database results.

In addition, we performed RT-qPCR and western blotting in several HCC cell lines and one immortalized hepatic cell line LO2. The results showed that MFSD2A expression was significantly lower in HCC cells than in LO2 cells (*P* < 0.05, Figure 1E, 1F).

### Downregulation of MFSD2A was correlated with poor differentiation in HCC

**Figure 2:**
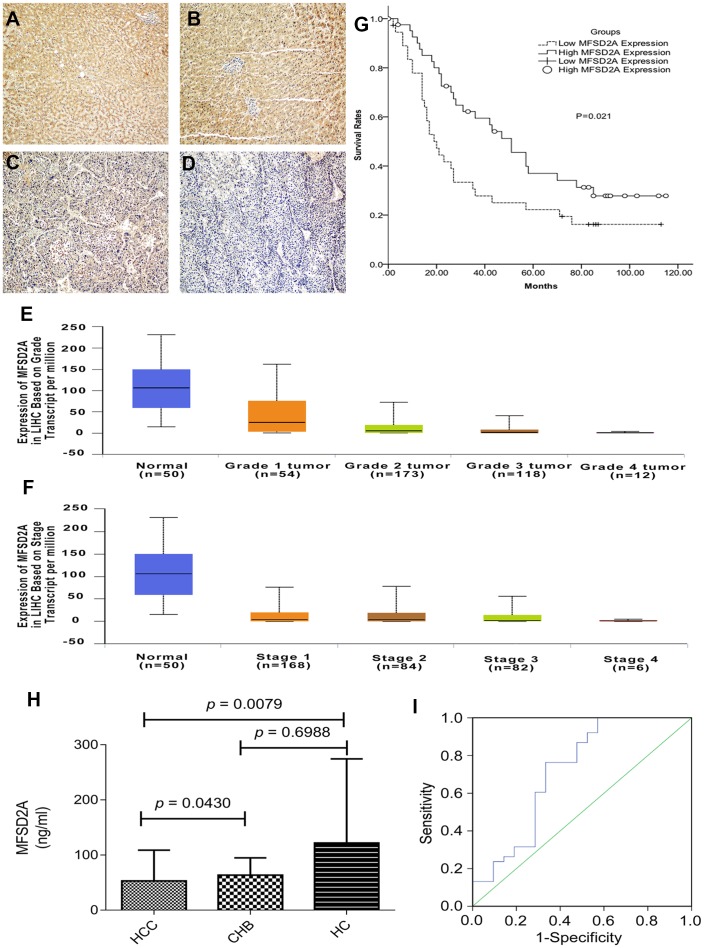
**MFSD2A expression and clinical values.** (**A**, **B**) High expression level of MFSD2A (200×magnification); (**C**) low expression level of MFSD2A (200×magnification); (**D**) negative expression of MFSD2A (200×magnification). Low expression of MFSD2A was significantly correlated with tumor grade (**E**) and stage (**F**) analyzed with UALCAN database. The lower expression level of MFSD2A represented higher stage and pathological grade. (**G**) Patients expressing higher levels of MFSD2A show significantly better five-year overall survival (*P* = 0.021). Survival curves of 79 HCC patients with different MFSD2A expression are shown. Kaplan-Meier survival curves for high expression of MFSD2A group were significantly different (*P*=0.021, log-rank test) from low MFSD2A expression group in 79 HCC patients. Diagnostic outcomes for plasma MFSD2A in the diagnosis of HCC (**H**) MFSD2A concentrations in plasma. (**I**) ROC curve for MFSD2A with HCC versus HC and CHB. MFSD2A=Major Facilitator Superfamily Domain Containing 2A, HCC=hepatocellular carcinoma, CHB=chronic hepatitis B virus infection, HC=healthy control.

Paraffin-embedded samples were obtained from 79 HCC patients after surgical resection. The patients had not received anticancer treatment prior to surgery (Table 1). We performed immunohistochemical staining on 79 paraffin-embedded HCC tissues. Overall, MFSD2A was positively and negatively expressed in 71 (89.9%) and 8 (10.1%) of the 79 HCC patients, respectively (Figure 2A–2C, total score ≥ 1; Figure 2D, total score = 0). In addition, 37 of 79 (46.8%) cases had low MFSD2A expression, whereas 42 (53.1%) cases had high expression (Figure 2A and 2B, total score ≥ 4; Figure 2C and 2D, total score < 4). As listed in Table 2, the low expression of MFSD2A was significantly correlated with poor histological differentiation (*P* = 0.012), but not with age, sex, tumor size, live cirrhosis, lymph node metastasis, recurrence, serum AFP level, or HBsAg status. Furthermore, we mapped the expression of MFSD2A in the UALCAN database and found that low expression of MFSD2A was significantly correlated with tumor grade and stage (*P*<0.05, Figure 2E, 2F).

**Table 1:** Clinical characteristics of 114 patients with hepatocellular carcinoma.

**Variables**	**N (%)**
Age	
>60	60(53.63)
<60	54(47.37)
Sex	
M	100(87.72)
F	14(12.28)
Tumor size	
>5cm	67(58.77)
<5cm	47(41.23)
Histological differentiation	
well	44(38.60)
moderate	37(32.46)
poor	33(28.95)
Liver cirrhosis	
Yes	95(83.33)
No	19(16.67)
HBsAg status	
positive	107(93.86)
negative	7(6.14)
Serum AFP	
positive	68(59.65)
negative	43(40.35)

HBsAg: Hepatitis B surface antigen; AFP: α-fetoprotein; N: numbers.

**Table 2:** Relationship between MFSD2A expression and clinicopathological features of hepatocellular carcinoma patients.

**Variables**	**MFSD2A**		***P* value**
	**low**	**high**	
Age			0.653
>60	16	21	
<60	21	21	
Sex			0.727
M	32	38	
F	5	4	
Tumor size			0.819
>5cm	14	18	
<5cm	23	24	
Histological differentiation			0.012^a^
well	10	23	
moderate	18	8	
poor	9	11	
Liver cirrhosis			0.576
Yes	31	32	
No	6	10	
Metastasis			0.364
Yes	1	4	
No	36	38	
Recurrence			0.463
Yes	5	3	
No	32	39	
HBsAg status			1.000
positive	34	39	
negative	3	3	
Serum AFP			0.317
positive	29	28	
negative	8	14	

MFSD2A: major facilitator superfamily domain containing 2.

HBsAg: Hepatitis B surface antigen; AFP: α-fetoprotein; N: numbers.

^a^P value<0.05.

### MFSD2A expression was an independent predictor of poor prognosis in HCC

To identify the variables of potential prognostic significance in all patients with HCC, we carried out univariate and multivariate analyses. Univariate analysis showed that MFSD2A expression, sex, and differentiation grade were significantly correlated with prognosis. MFSD2A expression was also an independent predictive factor for prognosis (*P* = 0.027) and differentiation grade (*P* = 0.013) based on multivariate analysis. The hazard ratio in patients with high levels of MFSD2A was 0.549 compared to that in patients with low expression (Table 3).

**Table 3:** Univariate and multivariate analyses showing the overall survival rate for hepatocellular patients.

**Variables**	**Univariate analysis**			**Multivariate analyses**		
	**HR**	**95% CI**	**P value**	**HR**	**95% CI**	**P value**
MFSD2A expression (high vs. low)	0.549	0.325-0.926	0.025a	0.549	0.323-0.935	0.027a
Age (>60 y vs. <60 y)	1.005	0.593-1.697	0.984			
Gender (male vs. female)	2.386	1.010-5.637	0.047a	1.801	0.718-4.515	1.801
Tumor size (≥5 cm vs. <5 cm)	1.390	0.817-2.365	0.224			
Histological differentiation (well vs. moderate vs. poor)	1.698	1.213-2.388	0.002a	1.604	1.105-2.330	0.013a
Cirrhosis (Y vs. N)	1.288	0.650-2.553	0.468			
HBsAg status (positive vs. negative)	0.872	0.348-2.186	0.770			
Serum AFP (positive vs. negative)	1.145	0.635-2.067	0.652			
Metastasis (Y vs. N)	1.442	0.575-3.619	0.435			
Recurrence (Y vs. N)	1.823	0.856-3.882	0.119			

MFSD2A: major facilitator superfamily domain containing 2; Y: yes; N: no; HR: hazard ratio; CI: confidence interval; HBsAg: Hepatitis B surface antigen; AFP: α-fetoprotein; N: number.

^a^*P* value<0.05.

In addition, the prognostic value of MFSD2A expression on HCC patient survival was evaluated between patients with high and low MFSD2A expression. The five-year overall survival in the high MFSD2A expression group was significantly better than that in the low MFSD2A expression group (*P* =0.021) (Figure 2G).

### The plasma level of MFSD2A was an optimal biomarker of HCC

Blood samples from 21 HCC patients, 16 HBV-infected controls and 22 healthy controls (HCs) were obtained and analyzed by ELISA to test serum MFSD2A expression. The median MFSD2A level was 31.77 ng/ml (range, 5.40–183.66) in 21 HCC patients, 52.13 ng/ml (range, 24.12–129.13) in 16 HBV-infected controls and 52.38 ng/ml (range, 27.10–718.90) in 22 HCs. The serum MFSD2A level in HCC patients was significantly lower than that in in HCs (*P* = 0.0079) and HBV-infected controls (*P* = 0.0430), resulting in an AUC of 0.718 (0.567–0.869), a sensitivity of 76.3%, and a specificity of 66.7% with an optimal cut-off value of 35.23 ng/ml, as shown in Figure 2H, 2I.

## DISCUSSION

HCC is the fifth most common cancer worldwide, and it has a poor prognosis despite improved diagnosis and combined therapy [15]. One of the most promising treatments for HCC is targeted therapy, which may play an important role in tumor growth [16]. Therefore, it is important to identify reliable biomarkers. These biomarkers may provide us with useful prognostic information; more importantly, these biomarkers may be targets for HCC treatment.

MFSD2A is a 532-amino acid protein with 11 putative membrane-spanning hydrophobic domains and N- and C-terminal ends, with both terminals predicted to be intracellular. MFSD2A has been previously identified as a putative receptor for Syncytin-2 and to act as a transporter in humans; its encoding gene in located at chromosomal position 1q34.2; and it belongs to the major facilitator superfamily [17]. The transporter function of MFSD2A has been shown to play a tumor-suppressive function in non-small lung cancer [18]. To the best of our knowledge, there has been no research on the relation between the expression of MFSD2A and the diagnosis and prognosis of HCC. In the current study, we detected the expression of MFSD2A in plasma and tissue samples and analyzed its value for diagnosis and prognosis.

In this study, the detection of MFSD2A mRNA and protein expression levels in paired HCC and adjacent normal tissues indicated that both mRNA and protein levels of MFSD2A were significantly lower in cancerous tissues than in the adjacent normal tissues, which was consistent with the findings of Spinola M in non-small lung cancer.

Further, based on immunohistochemical staining results, MFSD2A was expressed in most HCC patients Low expression of MFSD2A was associated with differentiation grade, implying that MFSD2A might play an important role in the development of HCC. We also confirmed the results in plasma samples. MFSD2A expression in patients with HCC was significantly lower than that in HCs and HBV-infected controls, achieving an optimal cut-off value of 35.23 ng/ml. The sensitivity was 76.3%, and the specificity was 66.7% in the plasma. This result indicates the diagnostic value of MFSD2A for HCC.

Multivariate analysis also indicated that MFSD2A was an independent predictor of survival. Kaplan-Meier survival analysis showed that the 5-year OS in the high MFSD2A expression group was significantly better than that in the low MFSD2A expression group, implying that MFSD2A can be used to predict prognosis.

In summary, our results showed that MFSD2A expression is downregulated in hepatocellular carcinoma. We also found that MFSD2A expression is correlated with histological differentiation. Multivariate Cox proportional hazards analysis revealed that MFSD2A and histological differentiation are independent factors of the five-year overall survival probability. Our study suggests that the expression levels of MFSD2A can be an independent prognostic indicator in HCC patients. The molecular mechanisms for this correlation, which may help us better understand the role of MFSD2A in the development of HCC, remain to be elucidated.

## METHODS

### Patients and tissue specimens

Samples from a total of 114 HCC patients undergoing surgical resection at Sun Yat-sen University Cancer Center were included in this study, including 35 paired HCC and matched adjacent noncancerous tissues collected between February and September 2010 and 79 paraffin-embedded samples obtained between 2003 and 2005. The whole research protocol was in accordance with the Declaration of Helsinki and approved by the Review Board of the Sun Yat-sen University Cancer Center. Written informed consent was also obtained for the study.

### Clinical data collection

All of the research data collected included age, sex, AFP level, metastatic lymph nodes, tumor number, tumor size, and TNM stage. The tumor stage was determined according to the AJCC 7^th^ TNM staging system [15].

### Cell culture

The cell lines LO2, HepG2, BEL7402, Hep3B, SK-Hep1 (Chinese Academy of Science, Cell Biology of Shanghai Institute, Shanghai, China) were cultured in RPMI-1640 medium (Gibco, Carlsbad, CA, USA) containing 10% fetal bovine serum (FBS; Gibco; Thermo Fisher Scientific, Inc.) in a humidified chamber with 5% CO2 and 95% air at 37°C.

### Blood samples

Blood samples were obtained from 21 HCC patients, 16 chronic HBV-infected controls and 22 healthy controls (HCs). After blood coagulation, the supernatant, represented serum, was centrifuged after balancing (usually 3000 rpm, centrifuge for 5–10 minutes). The supernatant was carefully aspirated (with attention not to aspirate the cell components) and stored at -80°C for preservation.

### Immunohistochemistry

Formalin-fixed paraffin-embedded samples were cut to 5 μm thickness. Each tissue section was dewaxed and rehydrated with graded ethanol. For antigen recovery, slides were boiled in EDTA (1 mm; pH 8.0) for 15 minutes in a microwave oven. At room temperature, the endogenous peroxidase activity was blocked by 0.3% hydrogen peroxide solution for 10 minutes. After washing with PBS, the slide was incubated overnight with polyclonal goat anti-human MFSD2A antibody (Prosci Company, diluted 1/300) at 4°C. After washing three times in PBS, the slides were incubated with HRP-conjugated anti-goat secondary antibody (Beijing Zhongshan Jinqiao Biotechnology Co., Ltd.) for 30 minutes at room temperature. Immunostaining was performed using the Envision system and diaminobenzidine (Dako, Glostrup, Denmark). Finally, signals were generated with 3,3′-diaminobenzidine tetrachloride (DAB), and all sections were counterstained with hematoxylin. Under the same experimental conditions, the tissue sections in the negative control group were incubated without the anti-MFSD2A antibody for immune response. The total MFSD2A immunostaining score represented the sum of the positive rate and staining intensity of tumor cells. The positive rate was “0” (< 5%, negative), “1” (5–25%, sporadic), “2” (25–50%, focused), or “3” (>50%, diffuse). The staining intensity scores were “0” (no staining), “1” (weak staining), “2” (moderate staining) and “3” (strong staining). The final expression score of MFSD2A ranged from 0 to 9. We determined the expression levels of MFSD2A as follows: “-” (score 0–1), “+” (score 2–3), “+” (score 4–6) and “++” (score > 6).

### Extraction of RNA and quantitative real-time PCR

Following the manufacturer’s protocol, 2 μg extracted RNA (TRIzol reagent) was reverse-transcribed into first-strand cDNA by M-MLV Reverse Transcriptase (Promega). Then, the following primers were used to amplify MFSD2A and GAPDH by quantitative real-time PCR: MFSD2A forward: 5′-CTCCTGGCCATCATGCTCT-3′ and reverse: 5′-GGCCACCAAGATGAGAAA-3′; GAPDH forward: 5′-CTCCTCCTGTTCGACAGTCAGC-3′ and reverse: 5′-CCCAATACGACCAAATCCGTT-3′. Gene-specific amplification was carried out in an ABI 7900HT real-time PCR system (Life Technologies) using a 15 μl PCR mixture including cDNA and appropriate primers. The mixture was preheated for 10 minutes at 95°C, then amplified for 30 seconds in 45 cycles at 95°C and for 1 minute at 60°C. The melting curve was measured for 15 seconds at 95°C, 15 seconds at 60°C and 15 seconds at 95°C. The Ct value of each sample was used to calculate the relative expression of MFSD2A against GAPDH expression (2^-ΔCt^ method).

### Western blotting

Tissue samples were lysed in RIPA lysis buffer and harvested by centrifugation (12,000 rpm, 30 minutes) at 4°C. The collected protein was separated by electrophoresis in a 15% sodium dodecyl sulfate polyacrylamide gel and transferred onto a polyvinylidene fluoride membrane. The membrane was placed in 5% nonfat milk for 1 h to block nonspecific binding sites and was then incubated with a goat anti-human MFSD2A antibody (ProSci incorporated; Cat.No.:6025, dilution 1:1,000) at 4°C overnight. After washing four times in Tris-buffered saline with Tween-20, the membrane was probed with a horseradish peroxidase (HRP)-conjugated rabbit anti-goat IgG antibody (1:5,000, Proteintech Group) at 37°C for 60 minutes. After four washes, bands were detected with an enhanced chemiluminescence reagent. Band density was measured with ImageJ software (National Institutes of Health) and was standardized to the density of GAPDH (mouse anti-human GAPDH monoclonal antibody, Shanghai Kangchen).

### Detection of plasma MFSD2A

We moved the required number of wells from the kit (Signalway Antibody LLC, EK3646, USA) from the refrigerator 20 minutes before the experiment to equilibrate them at room temperature (20–25°C) and returned the remaining sealed wells to 4°C. A standard curve was established before adding samples: 8 wells were set as standard wells; 100 μl of sample dilution solution was added to each well; 100 μl of standard was added to one well, mixed thoroughly; and 100 μl was aspirated with a pipet and moved to the second well. Therefore, the mixture was repeatedly diluted to the seventh well, and 100 μl was removed from the seventh well and discarded so that the volume remained 100 μl. The eighth hole was the blank control; 100 μl of the sample was tested in each well of the test object. The reaction plate was incubated at 37°C for 120 minutes, washed thoroughly with washing solution 4–6 times, and dried on a filter paper; 50 μl antibody was added to the working solution per well; the resultant solution was mixed thoroughly, and the reaction plate was allowed to stand at 37°C for 60 minutes; the reaction plate was washed thoroughly with the washing solution 4–6 times, and dried on a filter paper; 100 μl of enzyme-labeled antibody working solution was added per well, and the reaction plate was incubated at 37°C for 60 minutes. The plate was washed again before adding 100 μl of the substrate solution to each well, and the reaction was continued for 5–10 minutes in the dark at 37°C. Finally, 50 μl of stop solution was added to each well and mixed before measuring the absorbance at 450 nm.

### Statistical analysis

Overall survival (OS) was defined from the time between the date of diagnosis to death or censoring of data. Differences in mRNA expression between HCC and noncancerous tissues were evaluated with paired-samples *t* tests, whereas protein expression levels were compared with paired Student’s *t* tests. The Kaplan-Meier method was used to compare survival using with the log-rank test. Univariate and multivariate analyses were performed to explore the effect of MFSD2A expression on patient survival. A two-sided *P* value < 0.05 was considered statistically significant. All statistical analyses were performed with SPSS software (version 16.0; SPSS Inc., Chicago, IL, USA).

### Ethics approval

The current study was approved by the Institutional Review Board of Sun Yat-sen University Cancer Center, and all procedures met the basic standards of the Declaration of Helsinki. Ethics, consent, and permissions: All patients were clearly informed of the potential risks and benefits before undergoing the procedure. All patients stated their understanding and signed the informed consent form voluntarily.

The authenticity of this article has been validated by uploading the key raw data onto the Research Data Deposit public platform, with the approval RDD number as RDDA2019001051 The datasets used and/or analyzed during the current study are also available from the corresponding author on reasonable request.
